# Effectiveness of Hemopatch® versus Surgicel® Original to control mild and moderate liver bleeding

**DOI:** 10.1186/s12893-022-01747-0

**Published:** 2022-08-14

**Authors:** Selman Uranues, Abraham Fingerhut, Eve Levin, Daniel Spazierer, Nastaran Rahimi, Bernhard Baumgartner

**Affiliations:** 1 Section for Surgical Research, Department of Surgery, Medical University of Graz, Graz, Austria; 2 Baxter Healthcare Corporation, Deerfield, IL USA; 3 Baxter Medical Products GmbH, Vienna, Austria; 4 Section for Surgical Research, Medical University of Graz, Auenbruggerplatz 29, 8036 Graz, Austria

**Keywords:** Hemostasis, Hemostat, Wound sealing, Hepatic bleeding, Liver, Patient safety, Surgery, Trauma, VIBe SCALE

## Abstract

**Background:**

Adjunct hemostats can be of use in certain surgical settings. We compared the effectiveness of two hemostats, Hemopatch® and Surgicel® Original in controlling bleeding from liver lesions in an experimental model.

**Methods:**

Control of grades 1 (mild) and 2 (moderate) bleeding (according to the Validated Intraoperative Bleeding [VIBe] SCALE) was assessed for 10 min after Hemopatch® (n = 198) or Surgicel® Original (n = 199) application on 397 liver surface lesions. The primary endpoint was hemostatic success (reaching VIBe SCALE grade 0 at 10 min). The secondary endpoint was time to hemostasis (time to reach and maintain grade 0). A generalized linear mixed model and an accelerated failure time model were used to assess the primary and secondary endpoints, respectively.

**Results:**

The overall hemostatic success rate of Hemopatch® was statistically significantly superior to that of Surgicel® Original (83.8% versus 73.4%; p = 0.0036; odds ratio [OR] 2.38, 95% confidence interval [CI] 1.33–4.27) and time to hemostasis was reduced by 15.9% (p = 0.0032; 95% CI 0.749–0.944). Grade 2 bleeds treated with Hemopatch® had statistically significantly higher hemostatic success (71.7% versus 48.5%; p = 0.0007; OR 2.97, 95% CI 1.58–5.58) and shorter time to hemostasis (49.6% reduction, p = 3.6 × 10^–8^); differences for grade 1 bleeds (hemostatic success rate or time to hemostasis) were not statistically significant.

**Conclusions:**

Hemopatch® provided better control of VIBe SCALE bleeding compared to Surgicel® Original for Grade 2 bleeds in this porcine model, highlighting the importance of choosing a suitable hemostat to optimize control of bleeding during surgery.

## Background

Management of bleeding is a critical outcome factor during surgical procedures [1]. Effective management of intraoperative and postoperative bleeding reduces the risk of complications, morbidity and mortality and treatment costs, especially those associated with blood and blood product transfusion [2]. The percentage of patients with bleeding-related complications varies by specialty, but overall, it can be as high as 28.5% in general and solid organ procedures and 47.4% in cardiac surgery [3]. Increasing numbers of cancer operations in more complex and advanced stages [4] and patients on anticoagulation and antiplatelet treatment may lead to a higher risk of intra- and postoperative bleeding [5, 6]. Similarly, bleeding management is also crucial when treating patients with visceral trauma and non-traumatic emergencies [7, 8]. Appropriately selected adjunct topical hemostats can help reduce the perioperative risk of bleeding [9] and therefore, might be useful, especially for the treatment of organ lacerations [10].

The purpose of this study was to evaluate the hemostatic effectiveness of two widely used hemostats, an *N*-hydroxysuccinimide functionalized polyethylene glycol-coated collagen patch (Hemopatch®, Baxter Healthcare Corporation, Deerfield, IL, USA) and an absorbable knitted oxidized regenerated cellulose fabric (Surgicel® Original, Ethicon Inc, Bridgewater NJ, USA), in Validated Intraoperative Bleeding (VIBe) SCALE [11] mild and moderate bleeding when applied to surgically induced liver lesions in a heparinized porcine model. Both hemostats are indicated for use in hepatobiliary procedures and are frequently used in this setting [12–18]. To our knowledge, there are currently no reports available that compare these adjunct hemostats using a surgeon validated bleeding scale to objectively determine effectiveness in specific bleeding severities.

## Methods

### Study design

Two hemostats were investigated: Hemopatch® and Surgicel® Original. Since adjunct hemostats have recently gained market approval for use in mild and moderate bleeding [19, 20], the treatment of VIBe SCALE grades 1 and 2 bleeding [14] was investigated.

Treatments were assigned to each target application site by a block randomization scheme created using the “blockrand” 17 package of the R Software (R Core Team, Austria, Vienna) [21, 22].

### Animals

Thirty-five healthy castrated male naïve Yorkshire cross swine (mean weight 55 ± 6.4 kg) were used. Animals were group housed in stainless steel pens on raised flooring and single housed for fasting, on a 12 h light/dark cycle. Temperature and humidity were kept within 18–25 °C and 30–70% ranges, respectively. Animals were fed a certified pig diet twice daily and were fasted overnight prior to surgery. Tap water was available ad libitum. All animals were observed daily for general health and signs of disease.

### Surgical procedure

Animals were anesthetized by veterinary anesthesiologists with intramuscular tiletamine/zolazepam (4.05–5.03/2.02–2.51 mg/kg), intramuscular xylazine (1.5–3.5 mg/kg), intravenous propofol (2–8 mg/kg) and isoflurane inhalation. Analgesia consisted of intramuscular buprenorphine (0.04–0.05 mg/kg) and lidocaine (0.5–2.0 mg/kg), administered subcutaneously along the incision.

Animals were placed in dorsal recumbency. A carotid artery catheter was placed for invasive blood pressure monitoring purposes, and a jugular vein line ensured fluid and supportive therapy administration as well as intraoperative blood sampling. A midline laparotomy was performed, and the liver was positioned for optimal exposure and maximal testing surface availability. Heparin was administered intravenously prior to lesion creation and throughout the hemostasis evaluation period as needed to maintain an activated clotting time of approximately twice that of baseline and a maximum of 600 s, thus creating a standardized impaired coagulation status across treatment groups [23]. Clotting time was measured at least every 25 min.

**Fig. 1:**
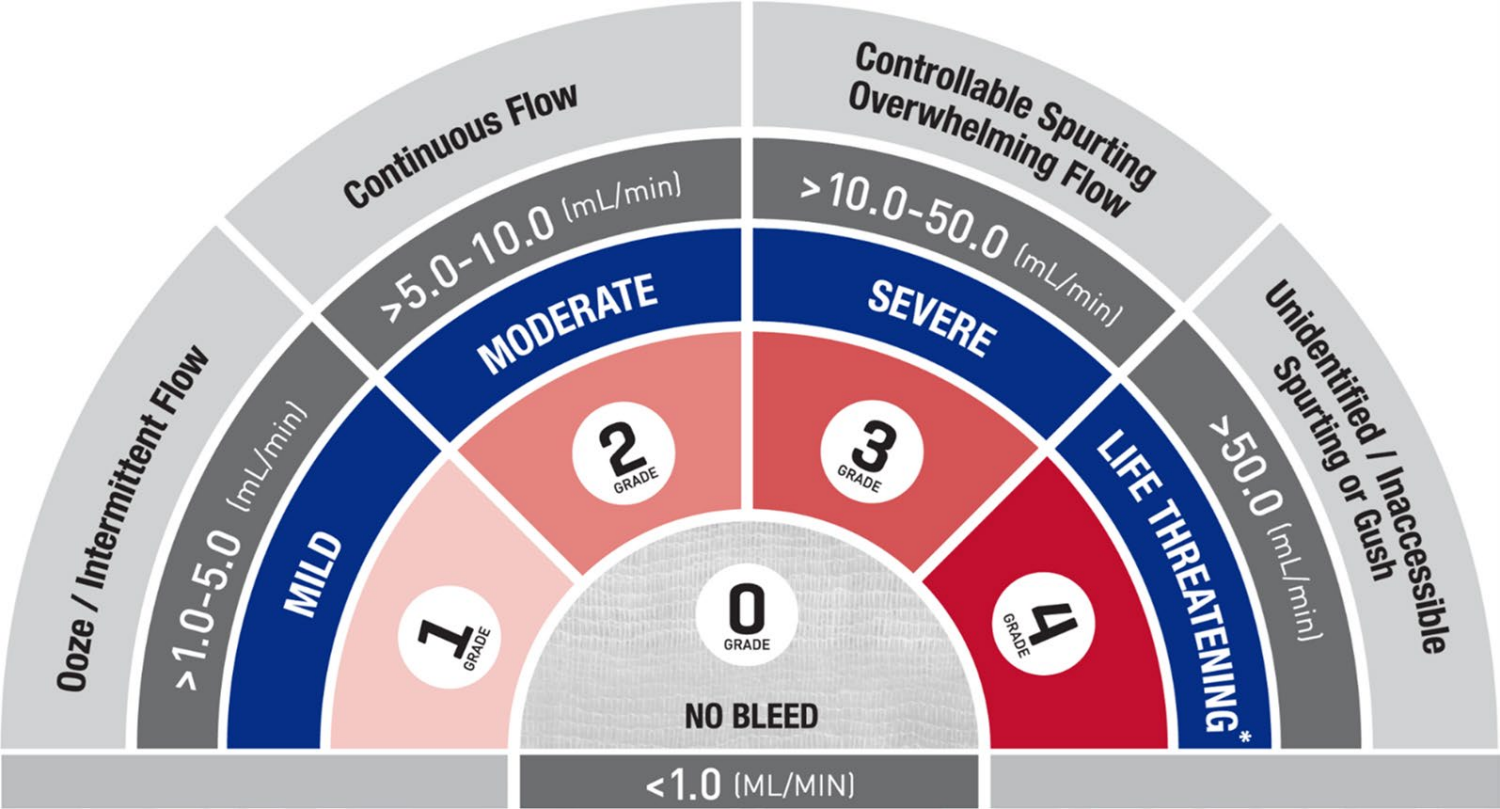
VIBe SCALE. *Systemic resuscitation was required (e.g., volume expanders, vasopressors, blood products, etc.). Adapted with permission of Elsevier Publishers at no cost to reproduce the VIBe SCALE table (in line with the BMC Surgery guidelines) from Surgery 161 (3): Lewis et al. [11]. Copyright Elsevier 2017

An electrocautery scratch pad was used to create lesions on the liver surface resulting in mild bleeding, which was defined as oozing or intermittent capillary-like bleeding at a rate of > 1.0–5.0 mL/min (VIBe SCALE grade 1; Fig. 1). Square lesions (approximately 1 × 1 cm, up to 4 mm deep) were created using sharp dissection. Liver tissue was removed using sharp and blunt dissection, maintaining the targeted depth within the squares to create moderate bleeding, which was defined as continuous flow bleeding at a rate of > 5.0–10.0 mL/min (VIBe SCALE grade 2; Fig. 1). Lesion pairs were created and assessed for 10 min after treatment prior to creating a new set of lesions. According to available anatomical space and animal physiological responses throughout the procedure, the median number of lesions per animal was 12. Only confirmed grades 1 or 2 bleeding lesions were included in the final analysis. All animals were euthanized after hemostasis evaluation.

### Assessment of bleeding

Bleeding was assessed by the study surgeon using the VIBe SCALE [11]. This scale classifies bleeding into 5 grades (ranging from 0: no bleed, to 4: life threatening) as shown in Fig. 1.

Pretreatment blood loss rate was determined quantitatively by measuring the amount of blood absorbed by a pre-weighed gauze in 10 s and calculating the blood loss rate per minute (1–5 mL/min for grade 1 and 5–10 mL/min for grade 2). Additionally, blood loss rate was measured for each treatment 10 min after Hemopatch® or Surgicel® Original application to determine whether hemostasis was achieved (VIBe SCALE grade 0, ≤ 1 mL/min). Potential bias was controlled by blinding the study surgeon to the treatment arm until the lesion was created and the VIBe SCALE grade was determined. Additionally, blood loss rates prior to treatment and at the primary endpoint assessment were quantified to confirm VIBe SCALE grades. Each treatment arm evaluated the effectiveness of Hemopatch® and Surgicel® Original for treating grades 1 and 2 bleeding combined, as well as separately.

### Application methods

Both Hemopatch® and Surgicel® Original were applied in single layers to the bleeding sites, overlapping the bleeding surface by at least 1 cm in accordance with their respective instructions for use [24, 25]. If hemostasis was not achieved at the pre-determined time points, additional pressure with gauze for 30-s intervals was allowed but no additional Hemopatch® was applied during the 10-min hemostasis evaluation period. For VIBe SCALE grade 2 bleeding sites, if hemostasis was not achieved at one of the pre-determined time points (2, 3, 5, and 7 min after application) a second layer of Surgicel® Original could be applied (for a total of two layers), followed by additional pressure/approximation using dry gauze for 30-s intervals, or until the next pre-determined evaluation time point.

### Study endpoints

The primary endpoint was hemostatic success, defined as achieving VIBe SCALE grade 0 (Fig. 1) 10 min after application. An observation period of 10 min was selected to include the anticipated time to hemostasis for the adjunct hemostats as well as a clinically acceptable intraoperative period to monitor and assure maintenance of hemostasis. The secondary endpoint was time to hemostasis, defined as the time post-treatment that the treated lesion reached and maintained grade 0. Lesions were evaluated at 2, 3, 5, 7 and 10 min after the start of treatment.

### Statistical analysis

A sample size of 197 lesions per treatment (a total of 394 lesions) was calculated to provide 80% power to detect a difference of 0.10 (calculated using PASS 2019, NCSS, East Kaysville, UT, USA). Logistic generalized linear mixed models were used to evaluate hemostatic success for overall (VIBe SCALE grades 1 and 2 bleeding combined), grade 1 bleeding, and grade 2 bleeding. The model included hemostatic success as the dependent variable, treatment as a fixed variable, pretreatment (baseline) bleeding grade as a fixed covariate (for overall bleeding only), and animal as a random effect. For analysis of time to hemostasis, an accelerated failure time model was used to model the difference in time to hemostasis between Hemopatch® and Surgicel® Original. The overall model included the fixed variables treatment and baseline bleeding grade (grade 1 or grade 2 bleeding), and animal as a random effect. Kaplan–Meier estimates of the median times to hemostasis between the two products were compared by the log-rank test. R software version 3.6.1 [22] was used to perform all analyses. P-values less than or equal to 0.05 were considered statistically significant.

## Results

Hemostatic effectiveness was evaluated on 198 and 199 lesions treated with Hemopatch® and Surgicel® Original, respectively. Pretreatment blood loss rates ranged from 1.02–4.74 to 5.04–9.96 mL/min for VIBe SCALE grade 1 and grade 2, respectively.

### Hemostatic success

The hemostatic success of Hemopatch® at 10 min (primary endpoint) was statistically significantly superior to that of Surgicel® Original for VIBe SCALE grades 1 and 2 combined (166/198 [83.8%] versus 146/199 [73.4%]; p = 0.0036; odds ratio [OR] 2.38, 95% confidence interval [CI] 1.33–4.27). In addition, Hemopatch® demonstrated greater hemostatic effectiveness at 2, 3, 5, and 7 min (Table 1).

**Table 1:** Hemostatic success of Hemopatch® and Surgicel® Original in VIBe SCALE grade 1 and grade 2 bleeding during the 10-min observation period post-application

Time (min)	Overall (VIBe SCALE grade 1/2)		VIBe SCALE grade 1		VIBe SCALE grade 2	
	Hemopatch® (N = 198)	Surgicel® Original (N = 199)	Hemopatch® (N = 99)	Surgicel® Original (N = 100)	Hemopatch® (N = 99)	Surgicel® Original (N = 99)
	n (%)	n (%)	n (%)	n (%)	n (%)	n (%)
2	142 (71.7)	101 (50.8)	94 (94.9)	95 (95.0)	48 (48.5)	6 (6.1)
3	146 (73.7)	107 (53.8)	94 (94.9)	95 (95.0)	52 (52.5)	12 (12.1)
5	153 (77.3)	122 (61.3)	94 (94.9)	95 (95.0)	59 (59.6)	27 (27.3)
7	159 (80.3)	131 (65.8)	94 (94.9)	95 (95.0)	65 (65.7)	36 (36.4)
10	166 (83.8)	146 (73.4)	95 (96.0)	98 (98.0)	71 (71.7)	48 (48.5)

For treatment of grade 1 bleeding, no statistically significant difference was found between the hemostatic success rates at 10 min for Hemopatch® and Surgicel® Original (95/99 [96.0%] versus 98/100 [98.0%]; p = 0.3396; OR 0.37, 95% CI 0.05–2.83). In contrast, for grade 2 bleeding the hemostatic success rate at 10 min for Hemopatch® was statistically significantly higher than that of Surgicel® Original (71/99 [71.7%] versus 48/99 [48.5%]; p = 0.0007; OR 2.97, 95% CI 1.58–5.58).

For VIBe SCALE bleeding grade 2, only 6.1% (6/99) lesions treated with a single layer of Surgicel® Original achieved hemostasis. For the remaining lesions an additional layer of Surgicel® Original was applied, of which 54.8% (51/93) still did not achieve hemostasis within the 10-min observation period.

### Time to hemostasis

**Fig. 2:**
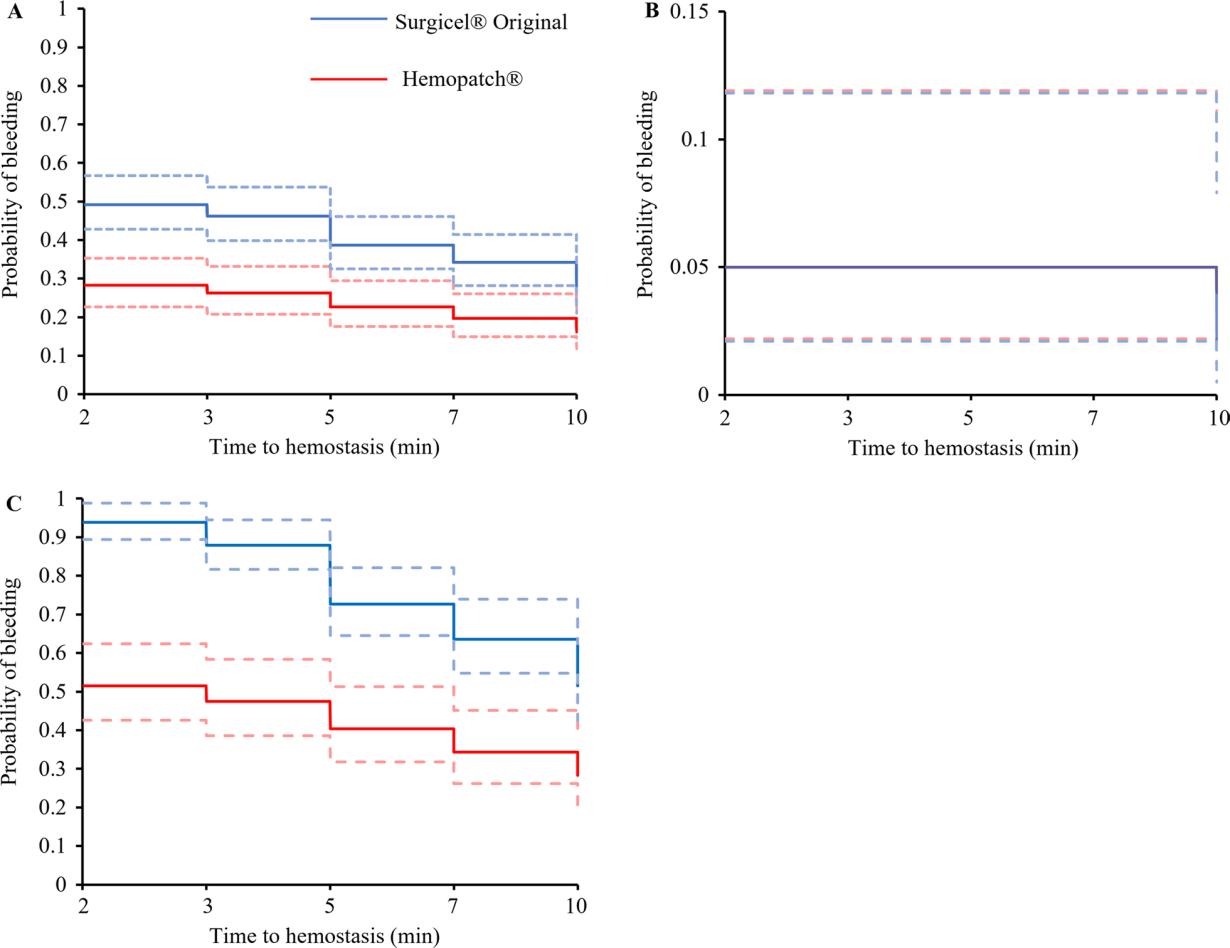
Kaplan–Meier plots of time to hemostasis: Hemopatch® and Surgicel® Original bleeding curves. **A** Overall bleeding curves for VIBe SCALE grade 1 and grade 2 bleeding combined. **B** VIBe SCALE grade 1 bleeding. **C** VIBe SCALE grade 2 bleeding. Dashed lines indicate 95% confidence intervals

For treatment of VIBe SCALE grades 1 and 2 bleeding combined, the time to hemostasis when using Hemopatch® was 15.9% shorter than that of Surgicel® Original (p = 0.0032; fold-change 0.841, 95% CI 0.749–0.944). The median time to hemostasis for combined grades 1 and 2 bleeding was 2 min for both the Hemopatch® and Surgicel® Original treatments (Table 2); however, the 95% CI for the median was narrower for Hemopatch® [2–2 min] than that for Surgicel® Original [2–5 min]. The probability of continued bleeding was statistically significantly lower for Hemopatch® than for Surgicel® Original at every time point other than 10 min (as shown by the non-overlapping 95% CIs; Fig. 2A).

**Table 2:** Kaplan–Meier estimates of median time to hemostasis

	Overall^a^		VIBe SCALE grade 1 bleeding		VIBe SCALE grade 2 bleeding	
	Median (min)	95% CI	Median (min)	95% CI	Median (min)	95% CI
Hemopatch®	2 (n = 198)	[2, 2]	2 (n = 99)	[2, 2]	3 (n = 99)	[2, 7]
Surgicel® Original	2 (n = 199)	[2, 5]	2 (n = 100)	[2, 2]	NA^b^ (n = 99)	[10, NA^b^]
P-value of difference in bleeding curves	0.0013		0.5574		9.33 × 10^−^^6^	

^a^VIBe SCALE grade 1 and grade 2 bleeding combined

^b^Could not be computed because less than half of the lesions had achieved hemostasis

For treatments of VIBe SCALE grades 1 and 2 bleeding evaluated separately, the time to hemostasis when using Hemopatch® was not statistically significantly different than that of Surgicel® Original for grade 1 bleeding (p = 0.51; fold-change 1.023, 95% CI 0.956–1.096); however, for grade 2 bleeding the time to hemostasis when using Hemopatch® was approximately half that of Surgicel® Original (p = 3.6 × 10^–8^; fold-change 0.504, 95% CI 0.395–0.644). The median time to hemostasis for grade 1 bleeding for both treatments was 2 min (95% CI 2–2 min) (Table 2). For grade 1 bleeding, there was no statistically significant difference in the probability of continued bleeding at all time points between the two hemostats (Fig. 2B). The median time to hemostasis for grade 2 bleeding treated with Hemopatch® was 3 min (95% CI 2–7 min). The median time to hemostasis and 95% CI for grade 2 bleeding treated with Surgicel® Original could not be computed because more than 50% of the grade 2 lesions did not achieve hemostasis (Table 2). The probability of continued bleeding of grade 2 lesions was statistically significantly lower for lesions treated with Hemopatch® than for those treated with Surgicel® Original at all time points (as shown by the non-overlapping 95% CIs; Fig. 2C).

## Discussion

This study showed that overall, using Hemopatch® to treat VIBe SCALE-defined mild and moderate bleeding in this heparinized porcine hepatic bleeding model increased (statistically significantly for grade 2 bleeding) the hemostatic success rate and resulted in statistically significantly shorter time to hemostasis compared to Surgicel® Original.

Given the increasing number of patients receiving anticoagulant prescriptions for long-term management of cardiac, cerebrovascular or peripheral vascular conditions [26, 27], surgeons frequently encounter patients taking these medications in both elective and emergency surgery settings, possibly leading to increased blood loss, longer operative times, greater use of surgical consumables, and higher costs [2]. Heparinization was therefore used in this study to mimic these clinical scenarios as well as intraoperative anticoagulation of patients. Active hemostats, such as Hemopatch®, are effective in patients with impaired coagulation, such as those receiving anticoagulation or antiplatelet therapies [28], whereas the efficacy of passive hemostats, such as Surgicel® Original is reduced in these patients [9, 28].

While a network meta-analysis study did not show that general use of local hemostatic agents reduces the rate of clinically relevant bleeding in thyroid surgery [29], a retrospective analysis of cardiac, vascular, noncardiac thoracic, solid organ, general reproductive organ, knee/hip replacement, spinal, and neurosurgical data from the Premier US Perspective Hospital Database reported fewer bleeding-related complications and shorter hospital/intensive care unit stays across all specialties when surgical bleeding was treated immediately with active hemostats, compared with treatment using a combination of passive and active hemostats [30], in which the passive hemostat is typically employed first. Combined with the results of a previous study of severe aortic bleeding [31], our results suggest that Hemopatch® is effective at achieving hemostasis across a broad range of bleeding severities. Taken together, this suggests that an active hemostat treatment strategy may be preferable across a range of surgical settings and where passive hemostats are less advisable based on their mechanism of action [32, 33].

One of the possible explanations we did not find any statistically significant difference in Grade 1 bleeding might be that manual pressure alone is sufficient in some instances of mild bleeding.

Given the increasing importance of bleeding management during surgery, discussed above, it is necessary to obtain objective evidence of a hemostat’s effectiveness in different settings and bleeding severities to enable surgeons to make appropriate treatment decisions. Semiquantitative scoring, quantification of the blood loss rate, or visual description of bleeding are frequently used in pre-clinical [32] and clinical settings [14, 33, 34], but these methods lack standardization across investigators and studies. Utilizing the VIBe SCALE helps to overcome these limitations as it is surgeon validated and can be easily interpreted across a broad range of surgical specialties [11].

The main limitation of this animal study is the proof of the clinical relevance of the results, although the porcine hepatic bleeding model is an accepted method for assessing performance of adjunct hemostats that have since been approved for use in patients [35, 36]. Hemopatch® is a thin, pliable collagen patch with NHS-PEG coating to facilitate sealing and hemostasis, which may provide an advantage for bleeding on the liver surface. Physical properties and practicality should be considered when selecting an adjunct topical hemostat for different clinical scenarios.

## Conclusions

This study showed that treating VIBe SCALE-defined mild and moderate bleeding with Hemopatch® increased the hemostatic success rate and decreased bleeding times compared to Surgicel® Original. In the context of increasing anticoagulant use and the evolving complexity of surgical procedures, the results of this study highlight the importance of choosing a suitable hemostat, whenever applicable, to optimize hemostasis during surgery.

## Data Availability

The data that support the findings of this study are available from Baxter Healthcare Corporation but restrictions apply to the availability of these data, which were used under license for the current study, and so are not publicly available. Data are however available from the authors upon reasonable request and with permission of Baxter Healthcare Corporation.

## Abbreviations

CI: Confidence interval
OR: Odds ratio
VIBe SCALE: Validated Intraoperative Bleeding SCALE
